# Genetic monogamy despite variable ecological conditions and social environment in the cooperatively breeding apostlebird

**DOI:** 10.1002/ece3.844

**Published:** 2013-10-28

**Authors:** Miyako H Warrington, Lee Ann Rollins, Nichola J Raihani, Andrew F Russell, Simon C Griffith

**Affiliations:** 1 Department of Biological Sciences, Macquarie UniversitySydney, New South Wales, 2109, Australia; 2 Genetics, Evolution and Environment, University College LondonGower St, London, WC1E 6BT, U.K; 3 Centre for Ecology & Conservation, College of Life & Environmental Sciences, University of ExeterPenryn, TR10 9EZ, Cornwall, U.K; 4 School of Biological, Earth and Environmental Sciences, University of New South WalesSydney, New South Wales, 2052, Australia

**Keywords:** Avian, extrapair paternity, polyandry, polygynandry, polygyny, reproductive flexibility

## Abstract

Mating strategies may be context-dependent and may vary across ecological and social contexts, demonstrating the role of these factors in driving the variation in genetic polyandry within and among species. Here, we took a longitudinal approach across 5 years (2006–2010), to study the apostlebird *(Struthidea cinerea),* an Australian cooperatively breeding bird, whose reproduction is affected by ecological “boom and bust” cycles. Climatic variation drives variation in the social (i.e., group sizes, proportion of males and females) and ecological (i.e., plant and insect abundance) context in which mating occurs. By quantifying variation in both social and ecological factors and characterizing the genetic mating system across multiple years using a molecular parentage analysis, we found that the genetic mating strategy did not vary among years despite significant variation in rainfall, driving primary production, and insect abundance, and corresponding variation in social parameters such as breeding group size. Group sizes in 2010, an ecologically good year, were significantly smaller (mean = 5.8 ± 0.9, *n* = 16) than in the drought affected years, between 2006 and 2008, (mean = 9.1 ± 0.5, *n* = 63). Overall, apostlebirds were consistently monogamous with few cases of multiple maternity or paternity (8 of 78 nests) across all years.

## Introduction

There is dramatic variation in the degree of extra-pair parentage in socially monogamous birds (range = 0–95% of broods, median = 9.1%, from Griffith et al. 2002). Although most of the interspecific variation in the level of genetic polyandry in socially monogamous birds can be attributed to deep-rooted phylogenetic variation in life-history traits (Griffith et al. 2002), contemporary comparisons across multiple populations or years are suggested to provide insight into the social and ecological causes of this variation (e.g., Petrie and Lipsitch 1994; Slagsvold and Lifjeld 1994; Stutchbury and Morton 1995; Bjornstad and Lifjeld 1997). For example, both interspecific and interpopulation comparative analyses have revealed lower levels of extrapair paternity in island versus mainland populations (Griffith et al. 1999; Griffith 2000), which at least partly, can be explained by a combination of social and ecological parameters (Ockendon et al. 2009). More recently, another interspecific comparative analysis revealed that genetic polyandry across socially monogamous birds was related to the degree of variation in rainfall and temperature, which influences the resource base and the predictability of those resources over time (Botero and Rubenstein 2011). The interpretation of interspecific analyses, however, remains problematic due to skewed distributions of phylogenies across spatial scales; our confidence in such approaches would be enhanced if the results were mirrored at smaller scales (Cockburn 2003; Cockburn and Russell 2011).

Although a number of studies have examined the variation in genetic mating strategies in a single species across multiple years or populations (Griffith et al. 1999; Bouwman et al. 2006; Johannessen et al. 2011; Townsend et al. 2011), these studies seldom explained much of the variation in genetic polyandry. One explanation might be a limited amount of underlying variation in important social and ecological parameters, such as operational adult sex ratio or the resource base that will affect the cost of parental care.

Cooperative breeding species have been found across a wide range of taxa including mammals (Lukas and Clutton-Brock 2012) and birds (Cornwallis et al. 2010) and broadly describe species where there are more than the breeding male and female contributing alloparental care to offspring at a nest. These “extra” individuals, known as “helpers” or “auxiliaries”, assist with the rearing of offspring that are not their own (Cockburn 2004). Cooperatively breeding species living in ecologically stochastic environments are likely to provide an apt model system for testing the relationship between social and ecological factors and variation in genetic mating strategy for several reasons.

First, there are many proposed costs and benefits associated with being a helper, and thus, there are many factors that may be driving the evolutionary dynamics of cooperatively breeding systems. For this study, we focused on the direct reproductive benefits of helping (see Koenig and Dickinson 2004 for additional costs and benefits of helping). Subordinate group members may receive both direct and indirect reproductive benefits, and the mating system determines what breeding opportunities and routes to evolutionary fitness are available to all group members. In some species, subordinate helpers gain direct reproductive benefits by either egg laying in the nest (females) or cuckolding the primary male breeder (males) (e.g., Richardson et al. 2001; Williams 2004; Du and Lu 2009). In other species, helpers appear restricted to gaining only indirect kin selected reproductive benefits (e.g., Conrad et al. 1998; Maccoll and Hatchwell 2004; Townsend et al. 2011). In ecologically challenging years, reproduction of individuals may be more ecologically constrained than in “good” years (shortage of resources such as food and water), and both the cost of breeding (breeders may need more help) and helping (takes more effort and cost to help) may be greater (as reviewed in Heinsohn and Legge 1999). In such years, greater incentive to help may be necessary and may include shared reproduction (e.g., Rubenstein 2007a).

Second, in cooperative vertebrates, ecological variability (1) is known to have significant effects on social structure and group size (Ekman et al. 2004; Russell 2004) and (2) is expected to contribute to the proportion of subordinates gaining reproduction within the group (Emlen 1982; Magrath et al. 2004). By extension, such species are likely to provide a suitable opportunity to investigate the relationship between genetic polyandry and social or ecological variation. Indeed, there is some supporting evidence to show a link between ecology and mating system within populations of cooperative breeders. For example, in a longitudinal study of the cooperatively breeding superb starling, *Lamprotornis superbus*, extrapair paternity within the population varied between groups and was related to the degree of vegetation cover and grasshopper abundance (Rubenstein 2007a). In another example, prior to a severe drought, a stable population of white-winged choughs (*Corcorax melanorhamphos*) were monogamous, but after the drought, group fragmentation led to polyandry and polygynandry occurring in breeding units that were comprised of multiple factions of birds (Heinsohn et al. 2000). Like white-winged choughs (Rowley 1978), apostlebirds in our population exhibited a fission–fusion society with smaller groups during the breeding season that occupied smaller territories and larger winter aggregations that ranged over larger areas during the nonbreeding season. This system facilitated the exchange of group members, which may offer individuals the possibility of forming new breeding coalitions and new groups (Griesser et al. 2009). As such, changes in group structure may lead to changes in genetic mating strategy.

The broad aim of this study was to use a within-population approach in the apostlebird, *Struthidea cinerea*, to test the link between ecology and the mating system and to do so over markedly contrasting years. A previous study of the apostlebird conducted in open Eucalypt woodland in southeastern Australia suggests that this species is an obligate cooperative breeder, with no pair able to successfully fledge offspring without the aid of helpers (Woxvold and Magrath 2005). In that population, the majority of helpers were philopatric offspring remaining on their natal territory, although immigrants (12.6% of helpers) also augmented group membership (Woxvold 2004). Helpers were shown to increase group productivity but not the survival of other group members (Woxvold and Magrath 2005). Across the 3 years of this study, there does not appear to have been significant ecological or social variation in patterns of reproduction or social structure. By contrast, the arid zone of Australia is characterized by environmentally driven cycles of ecological boom and bust that are driven by long-term patterns of highly unpredictable and spatially and temporally heterogeneous rainfall (Morton et al. 2011).

In this paper, we report a longitudinal study of the genetic mating strategy in a population of apostlebirds breeding in the western and most arid part of the species' range in southeastern Australia. The rainfall conditions during the course of our study were extreme and included the worst inland drought recorded in a century and one of the strongest La Ninã events in a century with a very high annual rainfall.

Our study of this species in the arid zone provided the opportunity to (a) characterize the pattern of social and genetic mating and contrast this with an earlier study conducted in a less ecologically challenging and less variable environment and (b) investigate the extent to which variation in ecological and social parameters over time affected the genetic mating system in a single population. We predicted that in years with lower rainfall, the proportion of broods with multiple paternity and maternity would increase. In such years, which are more ecologically challenging, there are likely to be more adults in the population that are ecologically constrained and unable to breed independently and are potentially competing for alternative opportunities to produce some offspring. Second, helpers have a significant effect on offspring survival in the apostlebird (Woxvold 2004), and it might make sense for a breeding female to trade a share of direct reproduction for increased help at the nest (i.e., Burke et al. 1989; Rubenstein 2007a), and other benefits such as increased allocation of the breeder's energy toward nonprovisioning activities (Heinsohn 2004) and improved long-term reproductive success (Russell et al. 2007) and survival (Kingma et al. 2009)

## Methods

### Study species and population

**Figure 1:**
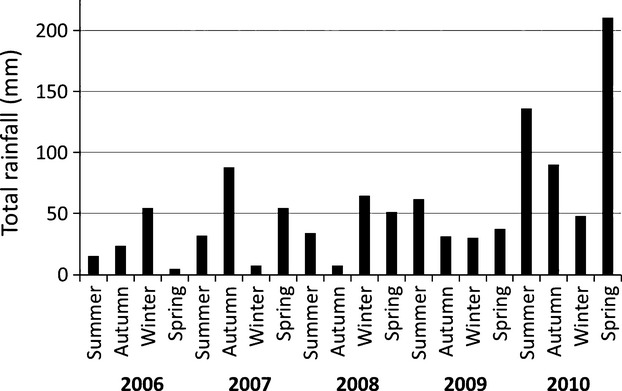
The seasonal distribution of rainfall at Fowlers Gap from 2006 to 2010. Summer is defined as from December to February, autumn is from March to May, winter is from June to August, and spring is from September to November.

**Figure 2:**
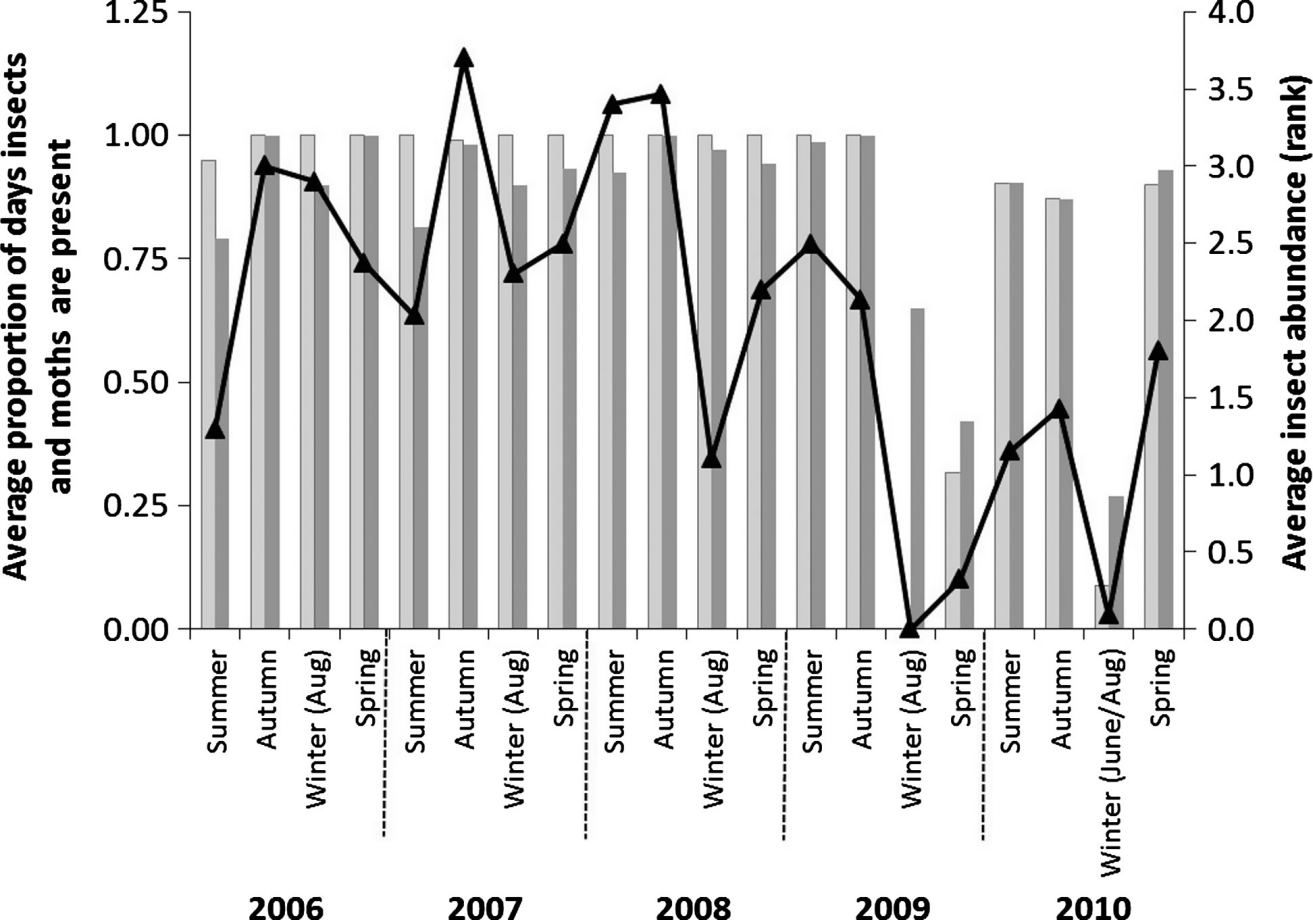
Seasonal insect presence at Fowlers Gap from 2006 to 2010. The left axis is the average proportion of days that insects (light gray) and moths (dark gray) were present. The right axis (solid line) is the average abundance of insects. Summer is defined as from December to February, autumn is from March to May, winter is from June to August, and spring is from September to November.

We studied the social organization of breeding apostlebirds at the Fowlers Gap Arid Zone Research Station (142°E 31°S, New South Wales, Australia) from 2006 to 2010. This study population has been monitored from 2004, and over 80% of resident adults in the study area have been individually color-banded with an unique combination of three color bands and a metal band (Australian Bird and Bat Banding Scheme). The climate at the study site is arid; the long-term average annual rainfall is 220 mm/year (all rainfall data from the Australian Bureau of Meteorology), and the pattern of rainfall is highly unpredictable with annual rainfall often falling in just two or three rain events with no seasonal pattern. Annual rainfall was 103 mm (2006), 208 mm (2007), 189 mm (2008), 126 mm (2009) during the dry years, while 2010 was a particularly wet year with 523 mm (Fig. 1). Daily insect data were collected at the Field Station as part of an ongoing monitoring program by the Australian Plague Locust Commission, and these data summarized the daily absence or presence of insects, as well as their relative abundance. Seasonal insect abundance remained high through 2006–2008 and dropped drastically in 2009 at the height of the drought with a slow recovery through 2010 (Fig. 2). Apostlebirds are omnivorous and adults feed their offspring predominantly insects (Higgins et al. 2006).

The study area straddles the Barrier Ranges, and the habitat is predominantly low open chenopod shrub land with small stands of the dominant trees *Acacia aneura* and *Casuarina pauper*. The habitat is also characterized by large expanses of bare ground, including bedrock of shale and quartzite, scree, gibber, and loose sandy clay. The only other dominant landscape feature is the large ephemeral desert creeks that typically run for just a few days in each year, but are lined with river red gums *Eucalyptus camaldulensis*. Apostlebird breeding territories are typically found alongside creek beds and artificial water bodies, as the birds rely on patches of mud to build their nests (Baldwin 1975).

The ecology of the Fowlers Gap field station is fairly typical of the Australian arid zone in that most animals and plants tend to follow an erratic pattern of “boom and bust” where good rains are followed by a dramatic increase in production but are interspersed by long dry periods of very limited production (Morton et al. 2011). Although the rains have no distinct seasonality, most resident birds in this area typically breed between August and December (the Austral spring) to presumably avoid the cool temperatures of winter and the hot temperatures of the summer.

### Field observations

We monitored the breeding activities of social groups (166 breeding attempts, defined as nests with ≥1 egg), over five seasons (August to December) from 2006 to 2010. However, because of insufficient monitoring in the peripheral parts of the study area with only 35.3% ± 7.55 of the birds in each group banded, we focused analyses of population and group size on the core area (124 breeding attempts, see Table 1), which is an area of approximately 25 km^2^ geographically defined by landmarks. This core area was consistently well sampled (90.2% ± 3.1 of birds banded) over the entire course of the study (2006–2010).

**Table 1:** Breeding ecology of the apostlebird: group numbers and sizes, nest productivity of all groups in the study population. Breeding season was determined by the earliest nest with an egg (start) to the last date of fledging (end)

Year	Breeding season	No. of groups	Mean distance to nearest neighbors' nest (m)	Group size range	Group size mean ± SE	No. of adult males mean ± SE	No. of adult females mean ± SE	No. of nest attempts	No. of failed nests	Mean no. of eggs/nest	Mean no. of fledglings/nest	Total no. of fledglings
2006	August–November	20	1031 ± 376	3–22	9.3 ± 1.1	5.0 ± 0.8	4.3 ± 0.6	18	1	3.9 ± 0.11	1.8 ± 0.28	45
2007	September–December	22	663 ± 113	4–22	8.6 ± 0.9	4.4 ± 0.6	4.6 ± 0.5	36	12	3.8 ± 0.19	1.5 ± 0.25	56
2008	August–December^a^	21	891 ± 137	5–15	9.3 ± 2.8	4.8 ± 0.4	4.1 ± 0.4	41	18	4.0 ± 0.16	1.3 ± 0.24	>49
2009	September	17^b^	2667 ± 334	5–20	10.8 ± 1.2	6.2 ± 0.8	4.8 ± 0.6	5	5	3.8 ± 0.20	0	0
2010	August–December^a^	16	746 ± 218	3–9	5.9 ± 0.5	3.3 ± 0.4	2.3 ± 0.3	24	6	4.3 ± 0.23	2.3 ± 0.37	49

a After the 2nd week of December when we ended the field season, groups were still breeding.

b Group sizes are not breeding group sizes, as only four groups made failed attempts at breeding, the other 13 groups made no attempts at breeding.

**Figure 3:**
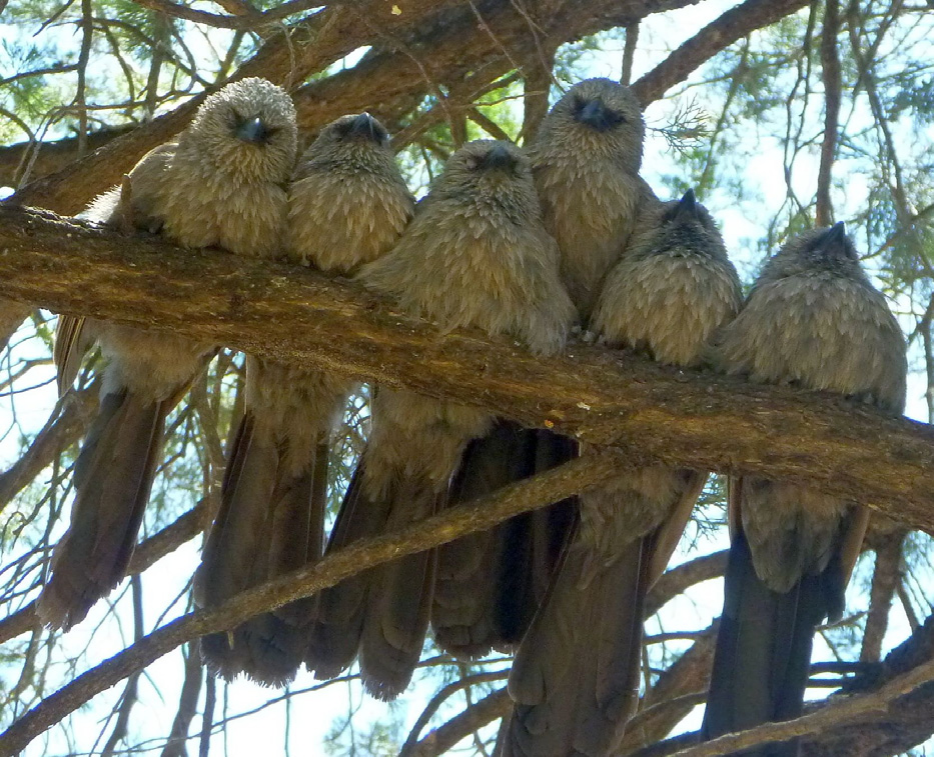
Adults in a social group of apostlebirds aggregating together.

Apostlebird breeding groups in our population ranged in size from 3 to 22 members and most were comprised of multiple males and females, which are highly social and aggregate together habitually (Fig. 3). Throughout periods of active breeding (i.e., time of first egg to fledging), group membership was monitored weekly, and all group members present at the nest and foraging sites were considered to be members of the group. Identifying the social breeding pair using behavioral cues was difficult and unreliable because although breeders often spent more time incubating and being present around the nest, there was no clear separation between the breeders and the more involved helpers as all adults contribute to all aspects of parental care and defense (Chapman 1998; Woxvold 2004; Woxvold and Magrath 2004, 2005; Woxvold et al. 2006). We thus refrained from defining putative parents behaviorally and when describing the genetic mating strategy, we referred to the rates of shared parentage (rather than extrapair rates) such that the genetic mating system is identified by the rates of polygyny (multiple maternity), polyandry (multiple paternity), and polygynandry (multiple paternity and maternity).

Although the number of eggs and nestlings was monitored using a mirror on a long pole, we only sampled young birds at the point of fledging due to the difficulty of accessing the nests. Young were banded with an unique combination of three color bands and a metal ring supplied by the Australian Bird and Bat Banding Scheme either before fledging, or within 1–3 days after fledgling (they are poor flyers for a few days immediately postfledging so could be caught by hand). In a few cases (7/198), young were caught by walk-in trap up to a few months after fledgling, but were still continuously associated with the group at the time of capture. Blood samples were taken from adults and offspring at the time of initial banding, and the sample (ca 30 μl) was collected via brachial venipuncture and stored in 1 ml of 95% ethanol.

### Molecular methods

DNA was extracted with a GentraPureGene DNA (Qiagen) extraction kit according to manufacturer's instructions. We amplified 20 species-specific microsatellite loci including *Sci1* (Woxvold et al. 2006); *Sci2*, *Sci4*, *Sci7*, *Sci8*, *Sci9*, *Sci10*, *Sci11*, *Sci12*, *Sci13*, *Sci14*, *Sci16,* and *Sci17* (Rollins et al. 2010) and seven new markers, *Sci18*, *Sci19*, *Sci21*, *Sci22*, *Sci26*, *Sci30,* and *Sci35* that were developed for this study (Table 2). The latter were developed using next-generation sequence data produced on the GS-FLX 454 platform (Roche, Germany) following methods described by Abdelkrim et al. (2009). Microsatellite repeat motifs were detected using QDD, v 0.9.0.0 Beta (Meglecz et al. 2010), and primers were designed using Primer 3 (Rozen and Skaletsky 1999). We used a Qiagen Multiplex Kit and a PCR program consisting of 95°C for 15 min; 35 cycles of 56°C for 30 sec, 72°C for 30 sec and 95°C for 30 sec; 56°C for 1 min; and 72°C for 30 min. Samples were genotyped on an ABI 3730 (Applied Biosystems, Foster City, CA) using GS-500 (Liz) in each capillary as a size standard. Allele sizes were estimated on GeneMapper, version 3.7 (Applied Biosystems). Microsatellite data from 76 breeding adults including no known relatives (parent–offspring sets) were tested for Hardy–Weinberg equilibrium using Arlequin, version 3.5.1.2 (Excoffier and Schneider 2005). Expected heterozygosity and the number of alleles per locus were calculated for this group of individuals using Arlequin. Loci were tested for evidence of linkage disequilibrium using Genepop, version 4.0 (Rousset 2008). *P-*values from all multiple comparisons were Bonferroni corrected. Five loci (*Sci1, Sci2, Sci4, Sci12,* and *Sci16*) had heterozygote deficits and significant departures from Hardy–Weinberg equilibrium. These loci and *Sci35* also showed consistent evidence of null alleles in reconstructed pedigrees and therefore were removed from further analyses. Therefore, for this study, we used the remaining 14 loci for parentage analysis in this study (Table 3) that had an average expected heterozygosity of 0.764 (± 0.120 S.D.) and 5–13 alleles per locus (mean = 7.6 ± 2.3 S.D.). All of these loci had low levels of null alleles and were in Hardy–Weinberg equilibrium. Sex was determined by molecular means using the *P2/P3* primer pair for polymerase chain reaction (PCR) amplification followed by digestion with *HAEIII* restriction enzyme (Griffiths et al. 1996), as other “universal” sex determining markers did not produce consistent results in this species.

**Table 2:** Description of seven variable microsatellite loci isolated from the Apostlebird (*Struthidea cinerea*). Seventy-eight breeding individuals were genotyped at each locus. For each locus, we list the repeat motif from the original sequence, forward and reverse primer sequences, allele size range in base pairs (bp), observed number of alleles (*N*_*A*_); observed heterozygosity (*H*_*O*_); expected heterozygosity (*H*_*E*_); and exact *P-*value of a test for deviations from Hardy–Weinberg equilibrium (none significant after sequential Bonferroni correction)

Locus/GenBank Accession	GenBank Accession number	Repeat Motif	Primer sequence (5′–3′)	Size range (bp)	*N*_*A*_	*H*_*O*_	*H*_*E*_	HW (exact) *P*-value
*Sci18*	JQ838038	(CCTAT)_17_	F: GCAGAGCTTAACTGATGCCC R: GCATGGAAAAGGGAAGATCA	233–278	9	0.821	0.828	0.272
*Sci19*	JQ838039	(ATCCC)_13_	F: CATGTGGGAACACAGTCCAG R: TGCTCCGTGGTGTGAGTATC	110–151	9	0.821	0.846	0.009
*Sci21*	JQ838040	(AC)_12_	F: GAAGTATCTCGGCCTTCCCT R: TTTCCCTGAAAGCTCTTGGA	104–124	6	0.436	0.382	0.661
*Sci22*	JQ838041	(TAT)_12_	F: TCATTGGGCTGTTAGGTTGTT R: GGCTGATGAATGAGGTGACA	137–185	13	0.885	0.876	0.152
*Sci26*	JQ838042	(CATCA)_10_	F: TTTGGTCCAGCACTGAAGAA R: CATGTCTGGATGACATTTTGCT	165–185	5	0.718	0.755	0.015
*Sci30*	JQ838043	(TA)_9_	F: TTCAGTTGTAAAGCAGGAGCC R: AAAACAAGAAAGGAAGAAAGAGAAAA	95–103	5	0.692	0.739	0.877
*Sci35*	JQ838044	(CT)_8_	F: TGAGGCCAGGGTAACAATTC R: GGTTGTTTTCCTAGGTTCGGA	169–177	5	0.654	0.665	0.282

**Table 3:** Primers used, absolute amount of primer per 5 μl reaction (picomoles), expected heterozygosity (*H*_*E*_), and number of alleles (*N*_*A*_). Mean and standard deviation (S.D.) are given for *H*_*E*_ and *N*_*A*_

Primer	Amount	*H*_*E*_	*N*_*A*_
*Sci7*	0.5 pM	0.728	6
*Sci8*	2.0 pM	0.854	10
*Sci9*	2.0 pM	0.715	10
*Sci10*	0.7 pM	0.792	6
*Sci11*	1.0 pM	0.768	7
*Sci13*	1.0 pM	0.822	8
*Sci14*	0.5 pM	0.786	7
*Sci17*	0.375 pM	0.797	6
*Sci18*	1.0 pM	0.829	9
*Sci19*	1.0 pM	0.846	9
*Sci21*	1.0 pM	0.382	6
*Sci22*	4.0 pM	0.876	13
*Sci26*	1.0 pM	0.755	5
*Sci30*	3.0 pM	0.739	5
Average (± SE)		0.764 (± 0.120)	7.6 (± 2.3)

### Reproductive strategy and assigning parentage

We assigned parentage using Cervus 3.0.3 (Kalinowski et al. 2007) and assumed an error rate of 0.01 in genotyping and that 90% of parents were sampled. Combined nonexclusion probabilities were calculated separately for each year of analysis (based on the adult population in that year) for all 14 loci. The combined nonexclusion probability for the first parent (Excl1) is the average probability of excluding an unrelated candidate parent from parentage when the genotype of the other parent is unknown (≤7.4E-04, for all years). The combined nonexclusion probability for the second parent (Excl2) is the average probability when the genotype of the other parent is known (≤5.5E-06, for all years). The combined nonexclusion probability for the parent pair (Excl3) is the average probability of excluding a pair of unrelated candidate parents (≤1.3E-09, for all years).

Apostlebirds appear to assume flexible and context depending mating strategies (Woxvold and Mulder 2008), and therefore, we used parent pair analyses so that all birds of possible breeding age (assumed to be at least a year of age, as there are no current studies on gonadal development or age of fecundity) in the whole population were included in analyses. These analyses allowed us to detect either a polygamous, polyandrous or monogamous mating strategy. All birds in the population, in each year that were not current offspring were considered as potential breeders, as well as the individuals with positive likelihood of descent (LOD) scores for each parent pair candidate for each offspring. Subsequent to the Cervus analyses, parents were confirmed based on manual checks of allelic matches to the offspring across the 14 loci. In most cases (159 of 180 offspring), assigned parent pairs had the highest LOD score and were therefore the most likely candidates. Most assigned parents (164 of 180 offspring) matched the offspring at all loci. In the 17 offspring that mismatched assigned parents, 13 offspring mismatched at one loci and four offspring mismatched at two loci (total of 21 mismatches, where 12 are consistent with allelic dropout and 9 appear to be genuine mismatches).

In those cases where more than one parent pair matched perfectly with the offspring (36 cases of parent matches for 24 of 180 offspring), all candidate parents were examined and the most parsimonious pair were selected on the basis of the following conditions all rejected pairs (*n* = 36) only matched one offspring in the entire brood, and one or both members of the alternate pair were A) not seen in the population despite group membership in previous years (*n* = 1); B) only observed in the population as a chick in past years (*n* = 4); C) the offspring's sibling from previous years (*n* = 12); D) the same sex as the other member in the parent pair (*n* = 11); E) sighted in another social group (*n* = 2); F) one year old (*n* = 1), all other breeding birds were between 3 and 5 years of age at first breeding (*n* = 9); or G) a female group member that only matched a portion of offspring, while another group female matched as mother for all offspring in the brood (*n* = 1). In four cases, an unbanded (and therefore unsampled) behaviorally dominant bird in the group was considered the more likely candidate, and the other candidate parent that matched only a portion of the entire brood was rejected as a parent.

In addition to the 24 offspring above, 56 offspring had zero mismatches with alternative candidate parents in addition to the individual we assigned as a parent (which also had no mismatches). In all these cases (98 parent matches to 56 of 180 offspring), the candidate parent matched no other individual in the population to form a parent pair that matched the offspring perfectly. Furthermore, the offspring was the only chick in the entire brood that matched the candidate parent, and the candidate parent was rejected on those grounds and the following conditions: the candidate parent was A) not sighted in the population (*n* = 37), B) only sighted in the past as a chick (*n* = 14, never sighted as an adult), C) a group bird with no candidate partner (adult, *n* = 12, yearling *n* = 10), D) a group bird that was an older sibling (*n* = 14), and E) sighted in a different group (*n* = 11).

### Statistical analysis

Logistic regressions were performed in R, version 2.15.1 (R Development Core Team 2012) using the package *‘aod’* (Lesnoff & Lancelot 2012) using the glm command followed by a Wald test. All other statistical tests were carried out in Minitab 16.2.2. We performed nonparametric tests (Kruskal Wallace, Mann–Whitney *U*-test) as our data were not normally distributed.

## Results

### Population size and breeding ecology

The estimated population size ranged from 149 to 199 individuals in each year across the period from 2006 to 2008. In the 2009 breeding season (at the height of the drought), when no groups successfully fledged offspring, the estimated population size dropped to 127 individuals, and by the 2010 breeding seasons, the population had dropped to 86 individuals. We excluded data from 2009 on apostlebird breeding group size, adult male numbers, and female numbers from analyses because few groups (5 nests, 1 group made two nest attempts) attempted to breed, while most birds remained in their larger nonbreeding social groups (see Table 1).

Mean breeding density, defined as the mean distance to the nearest neighbor's nest from 2006 to 2008 and 2010 did not vary significantly from each other (mean ± SE = 803.9 ± 100.8 meters; Kruskal–Wallis *H* = 3.19, *n* = 67, *P* = 0.36, Table 1). Breeding group sizes from 2006, 2007, and 2008 did not significantly vary from each other (mean ± SE = 9.1 ± 0.5, *n* = 63), while group sizes in 2010 were significantly smaller (mean ± SE = 5.9 ± 1.5, *n* = 16) than in groups in 2006–2008 (Kruskal–Wallis *H* = 13.01, *n* = 79, *P* = 0.005, Table 1; Mann–Whitney *W* = 450, 513, 512 and *P* = 0.01, 0.01, 0.0006 for 2006, 2007, and 2008 compared with 2010, respectively). The number of adult males in each group ranged from 1 to 13 males with an average of 4.4 ± 0.3 males per group (*n* = 68 groups) and did not significantly vary across 2006–2008 and 2010 (Kruskal–Wallis *H* = 6.2, *n* = 68, *P* = 0.10, Table 1). The number of adult females in each group ranged from 1 to 11 females and varied significantly among years (Kruskal–Wallis *H* = 13.9, *n* = 68, *P* = 0.003; Mann–Whitney *W* = 323, 390, 381 and *P* = 0.009, 0.002, 0.007 for 2006, 2007, and 2008 compared with 2010, respectively) with the number of females in each group being significantly smaller in 2010 (mean = 2.3 ± 0.3, *n* = 16) than in 2006–2008 (mean ± SE = 4.3 ± 0.3, *n* = 52, see Table 1).

Despite the change in population and breeding group sizes, roughly the same number of groups (16–22) were present in our field site from 2006 to 2010 (Table 1). The number of nest attempts across the whole population in each year varied from 5 to 36 attempts, with varying rates of nest failure (1–18 nests/5–100% of nests in each year). There was no significant variation in the clutch size (Kruskal–Wallis *H* = 2.88, *n* = 88 groups, *P* = 0.41, Table 1), the number of nestlings (Kruskal–Wallis *H* = 2.26, *n* = 85 groups, *P* = 0.52, Table 1), and the number of fledglings (Kruskal–Wallis *H* = 5.87, *n* = 88 groups, *P* = 0.12, Table 1) produced across 2006–2008 and 2010. Similar clutch sizes (mean = 3.99 ± 0.10 eggs/nest, nest = 88), nestlings (mean = 2.19 ± 0.17 nestlings/nest, nest = 85), and fledglings (mean = 1.61 ± 0.15 fledglings/nest, nest = 88) were produced in ecologically good years (2006–2008 and 2010) between August and December (Table 1).

The ratio of adult males to adult females in breeding groups was 1.57 ± 0.15 and did not significantly differ among the breeding years (Kruskal–Wallis *H* = 4.67, *n* = 54 groups, *P* = 0.20, Table 4). The proportion of males breeding within the field site was 0.34 ± 0.03 males/nest and did not significantly vary among years (Kruskal–Wallis *H* = 5.68, *n* = 54, *P* = 0.13, Table 4). The proportion of adult females that bred, however, was significantly higher in 2010 (Kruskal–Wallis *H* = 10.9, *n* = 54, *P* = 0.01; Mann–Whitney *W* = 117, 137, 157 and *P* = 0.003, 0.05, 0.006 for 2006, 2007, and 2008 compared with 2010, respectively; Table 4), with 16 of 33 females breeding (mean 0.63 ± 0.09 females per nest, *n* = 18 nests), in contrast to the years 2006–2008, when on average 15 ± 0.5 of 57 ± 0.35 females bred (mean = 0.33 ± 0.03 per nest, *n* = 41 nests).

**Table 4:** Number of breeding males and females, helping nonbreeding male and females in core groups where parentage was determined in >1 parents

Year	No. of nests with ≥1 parents sampled	No. of breeding females	No. of female helpers	Prop females that bred	No. of breeding males	No. of male helpers	Prop males that bred
2006	13	14	45	0.29 ± 0.03	14	54	0.32 ± 0.07
2007	21	16	40	0.38 ± 0.07	15	47	0.36 ± 0.09
2008	18	15	41	0.32 ± 0.03	16	61	0.26 ± 0.05
2010	18	16	17	0.63 ± 0.09	18	32	0.41 ± 0.06

### Parentage and reproductive skew

In most nests from 2006 to –2008 and 2010, all or most members of the group had been sampled (86.6% ± 3.3), and we determined parentage of 198 offspring from 86 nests. We were able to assign parentage to both parents from 72% (62 of 86) of nests in 2006–2008, and 2010, and parentage to one parent in a further 19% (16 of 86) of nests in 2006–2008, and 2010. In 9% of nests (8/86) in 2006–2008 and 2010, we were unable to assign any parentage, as neither genetic parent had been sampled. Cases of multiple maternity and paternity were few, occurring over the four breeding seasons in just 10% (8 of 78) of nests. The levels did not vary significantly between 2006 and 2008 and 2010 (Fisher's Exact Test two-tailed, *n* = 78, *P* = 0.09). In 70 of the 78 nests where one or both parents for each chick were identified, the parents were a genetically monogamous pair, 3% of nests (2 of 78) had multiple paternity (polyandry), and 3% of nests (2 of 78) had multiple maternity (polygyny). In 4% of nests (3 of 78), there were two sets of unique pairs from within the group (polygynandry) sharing parentage of the brood, and in one additional nest (1 of 78), one female mated monogamously with a male, while the other female mated polyandrously with the same male and a second new male (polygynandry) (Table 5).

**Table 5:** Genetic mating strategy of apostlebirds. Parentage is from groups where one or both parents were sampled. In three of the polygynandrous broods (2006, 2007, 2010), two males and two females gained parentage as two separate monogamous couples within one brood. In one brood in 2010, one female mated monogamously with one male, while a second female mated polyandrously with the same male plus another different male (two females and two males)

Year	No. of nests (broods)	No. of offspring	Monogamous broods	Polyandrous broods	Polygamous broods	Polygynandrous broods
2006	13	32	12	0	0	1
2007	21	48	18	0	2	1
2008	25	54	25	0	0	0
2010	19	46	15	2	0	2
Total	78	180	70	2	2	4

We pooled data from 2006 to 2008 and examined the 2010 data separately to determine the effects of group size on the mating strategy within a group. The following factors did not predict combined rates of polygyny, polyandry, and polygynandry from 2006 to 2010: group size (2006–2008, Logistic regression χ^2^ = 0.39, *n* = 41, *P* = 0.53; 2010 Logistic regression χ^2^ = 2.4, *n* = 13, *P* = 0.12), the number of adult males (2006–2008 & 2010, Logistic regression χ^2^ = 1.9, *n* = 54, *P* = 0.16), and the number of adult females, (2006–2008, Logistic regression χ^2^ = 0.1, *n* = 41, *P* = 0.75; 2010, Logistic regression χ^2^ = 3.2, *n* = 13, *P* = 0.07). Multiple paternity and maternity occurred in breeding groups ranging from 4 to 10 members.

In the eight polygamous, polyandrous, and polygynandrous nests, seven broods represented groups that had more than one nest that season. In all seven cases, shared reproduction occurred only in the second nest. In the one remaining nest, the social group only had one nest that season. All nests were started (eggs laid) from mid-September to mid-November. Of the 14 females that bred in polygamous, polyandrous, and/or polygynandrous nests, 57% were first time breeders.

## Discussion

Apostlebirds were largely monogamous across several years of differing ecological conditions that likely represent the ecological and social extremes of what the species faces. Although they exhibit the flexibility to employ differing mating strategies, such as polyandry, polygyny, and polygynandry, these different tactics did not vary across the ecological or social variation seen in our longitudinal study. The proportion of broods with multiple mothers or fathers was not influenced by the level of rainfall or insect presence and abundance, which we may have expected due to the potential value of increasing helper incentives at the nest during times of low insect abundance (such as in a trading sex for help strategy, see Rubenstein 2007b). We also expected that periods of low rainfall would coincide with low insect abundance; however, insect abundance was higher during the beginning of the study (when rainfall was lower) and decreased as the drought progressed. This demonstrated a time lag between weather conditions (rain) and other ecological conditions (such as availability of food sources). We discuss the effect of time lag later in this discussion. However, mean insect abundance still varied between the “drought” and “wet” periods and still we did not see variation in the genetic mating strategy. Furthermore, the number of eggs, nestlings, and fledglings produced (group productivity) across the years in which apostlebirds bred (2006–2008, 2010) were roughly the same despite decreased insect abundance in 2010. However, in 2009, when insect abundances were very low, few apostlebird groups made attempts to breed (Table 1).

**Figure 4:**
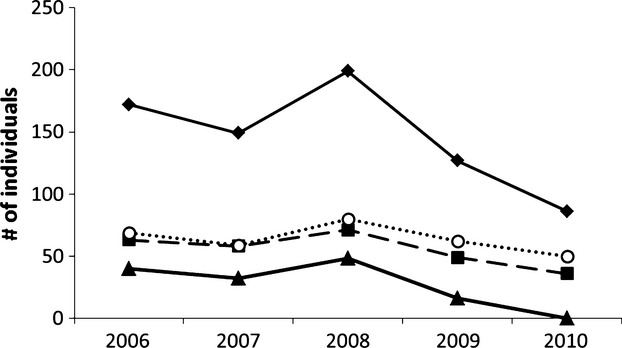
Estimated core population size of apostlebirds at Fowlers Gap. The solid line with solid filled diamond, 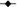, is the total number of individuals in the population. The dashed line with solid filled square, 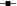, is the total number of known females and the dotted line with unfilled circle, 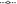, is the total number of known males. The total number of birds of unknown sex is the solid line with solid filled triangle, 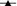.

We expected to see differing rates of multiple paternity and maternity in association with a change in breeding group size. Smaller group sizes can increase an individual's chance of breeding as seen in 2010, when a significantly higher proportion (0.62 ± 0.09) of females in the population bred than in 2006–2008 (0.33 ± 0.03). Smaller group sizes in 2010 may have been a result of a drastic decrease in the total number of birds found in the area (Fig. 4). In 2009, the population had dropped to 127 birds from 149 to 199 birds in previous years. Thus, the higher proportion of breeding females in 2010 was associated with smaller group sizes and fewer females in the population rather than an increase in polygyny.

The proportion of broods with multiple mothers or fathers was not influenced by total group size or the number of adult females or males, despite smaller group sizes in 2010 and lower numbers of adult females in 2010, following a year (2009) of unproductive breeding at the height of the drought. This contrasts to a population of the closely related white-winged chough, which reorganized social structure following an intense drought and a particularly harsh, cold winter (Heinsohn et al. 2000). In this chough population, group composition and reproductive skew changed, with increased rates of polyandry and polygynandry after the drought.

We did not observe a relationship between group productivity and genetic mating strategy because neither varied in our study, even though group sizes differed between the drought and wet periods. This was unexpected given that the previous study in a woodland population showed that helpers had a significant effect on offspring survival in the apostlebird (Woxvold 2004). Other factors may be driving group productivity in our arid zone population, or perhaps in drought years, more helpers are needed to maintain group productivity. However, this also demonstrates that females may not be trading a share of reproduction for more help, and rather incentives to help in apostlebirds may be other benefits (see later in discussion).

In addition to genetic mating strategy not being affected by large variation in rainfall and insect abundance at our arid zone study site (hereafter referred to arid zone, AZ), our results were also similar to those found in another population of the species breeding in a nonarid area of dry woodland (hereafter referred to as woodland, WL; Woxvold and Mulder 2008). Despite these ecological differences (WL mean annual rainfall = 405 mm/year, versus AZ mean annual rainfall = 220 mm/year), genetic mating strategy in the woodland population was similar to our arid zone population, with most groups employing a monogamous mating strategy (11/18 groups) and fewer groups (4/18 groups) exhibiting polygyny. Further, in the woodland site, group sizes were similar to group sizes in our study (WL mean = 7.8 ± 1.4, AZ mean ± SE = 9.1 ± 0.5 in 2006–2008, 5.9 ± 0.5 in 2010) and the number of females per group (WL mean = 2.4 ± 0.4) and the proportion of breeding females (WL mean = 0.71 ± 0.12) was similar to the number of females per group in 2010 (AZ mean = 2.3 ± 0.3) and the proportion of breeding females in 2010 (AZ mean = 0.63 ± 0.09) in our study. Also, the number of males per group in the woodland site (WL mean = 3.6 ± 1.1 males) was also similar to our study (AZ mean = 4.7 ± 0.3 males from 2006 to 2010). However, the proportion of breeding males in the woodland site (WL mean = 0.59 ± 0.12) was greater than in our population (AZ mean = 0.34 ± 0.03 males from 2006 to 2010). Overall, this suggests that genetic monogamy in this species is a characteristic that does not vary with group structure and habitat traits such as rainfall or insect abundance, even in the most extreme part of this species' range with respect to aridity and unpredictability of rainfall.

However, our population differed from the woodland population (Woxvold and Mulder 2008) in the distribution of breeding territories. In our population, breeding sites were located along creek beds, as well as surrounding the edges of two lakes. This linear arrangement of territories is in contrast with the more mosaic-like pattern of territories in the woodland study (Woxvold and Mulder 2008). Territory arrangement influences breeding density and the number of adjacent neighboring groups and hence may affect access to possible extrapair sires. It has been suggested that the linear arrangement of territories in purple-crowned fairy wren habitat explained the relatively low level of extrapair paternity in that population (Kingma et al. 2009). Likewise, apostlebirds in our study would have had fewer adjacent neighbors than those in the woodland population. However, clear differences in the rates of extrapair paternity between the two populations failed to emerge.

The rate of multiple paternity and maternity may be underestimated in our study as we only sampled fledglings. On average, the clutch size was 3.99 ± 0.10 eggs, while the average number of fledglings produced was 1.61 ± 0.15. Sampling of all eggs may have revealed a higher rate of multiple paternity. Furthermore, copulations were not observed during our study, and given that the relationship between the number of extrapair copulations and extrapair paternity is unlikely to be straightforward (Dunn and Lifjeld 1994; Griffith 2007), the level of actual multiple paternity underestimates the level of multiple mating by females.

Furthermore, measuring and interpreting within population variation in mating strategies is challenging because differences across time may be affected by temporal lags between different variables. The impact of changing ecological conditions (such as a decrease in food supply) may be manifested years later, so it is possible that the time frame of this study was insufficient to study the impact of ecological conditions on mating strategy. However, the immediate effects of ecological conditions, such as mortality and survival was manifested in variation in group size and composition that varied across years, so we can be reasonably confident that these factors did not seem to affect genetic mating strategy.

Interestingly, the number of females that bred within our population remained roughly the same over the years, suggesting that perhaps the study area may only have a certain number of breeding territories, and hence, ecological constraints may be an important factor for cooperative breeding in apostlebirds. However, we did not estimate breeding territory size, which may also likely have changed in size between the drought and wet periods. Previous research has shown that territory size may vary due to the availability of resources (food, access to water) and group size, and may also influence the rate of extrapair paternity within a population (i.e., Brooker and Rowley 1995). However, ecological constraints do not appear to be driving nonbreeding individuals to stay on a territory and help. A previous study on a woodland population of apostlebirds found low rates of dispersal (which may indicate ecological restraints), but also found evidence that birds remained on the natal territory despite the capability of independent breeding. Following nest failure, a group of birds split into two groups and subsequently both groups produced fledglings later in the season. Furthermore, several groups shifted breeding territories between broods within the same season, and many territories that were occupied during some years in the woodland study were found vacant in other years (Woxvold 2004). This is similar to what was observed in our study, when in 2010, one group with 12 adult birds produced a nest early in the season and then subsequently produced two simultaneous broods (which fledged 1 day apart) within 241 meters of each other. The exact split of adult birds was unknown; however, one of the two broods (at the original first brood site) had the same parents as the first brood of the season, while the other brood bred had four different parents (2 males and 2 females).

Furthermore, it also appears that the apostlebird's closest living relative, the white-winged chough, *Corcorax melanorhamphos,* which was also not restricted by ecological conditions, but rather by the long delay in learning foraging skills. Previous studies demonstrated that choughs needed at least two helpers to provision young at a sufficient rate to produce fledglings (Heinsohn et al. 1988) and that group productivity was positively related to the number of helpers (Boland et al. 1997) indicating that helpers were a crucial resource for breeding. So perhaps nonbreeding apostlebirds may also be restricted by their ability to obtain helpers, and more helpers are needed in ecologically “bad” years. However, even in the drought years, when apostlebird group sizes were larger, we did not observe more groups breeding. This does not necessarily indicate ecological constraint or restriction caused by group size, but perhaps an interplay between the available resources and the number of helpers it takes to successfully provision a brood. In the white-winged choughs, supplemental feeding of small groups resulted in smaller groups producing as many fledglings as larger groups (Boland et al. 1997), so perhaps in drought years, apostlebirds require more helpers to successfully fledge young, while in wet “good” years, smaller groups may be able to successfully fledge young. Future experimental feeding experiments and quantification of territory quality may elucidate the conditions driving helping behavior, number of territories, and group size in apostlebirds.

The costs and benefits of a long-term pair bond may also influence the rate of genetic monogamy. Longer pair bonds may allow for the accumulation of philopatric offspring helpers that derive indirect reproductive benefits from raising full siblings. For example, in one social group, group size increased from three birds in 2005 to 10 birds in 2009 solely via retained offspring. In many other species of cooperative breeders, (e.g., Conrad et al. 1998; Blackmore and Heinsohn 2007) helpers are largely philopatric young. Genetic monogamy may then be favored if the benefits of mate retention and long-term pair bonds, such as increased familiarity, compatibility, and reproductive fitness outweigh the costs of mate retention or changing mates [as reviewed in Ens et al. (1996)]. Furthermore, monogamy may be favored because it maximizes indirect fitness benefits of helping for retained offspring helpers (monogamy hypothesis, see Cornwallis et al. 2010).

In summary, apostlebirds were largely monogamous although they exhibited the flexibility to employ different mating strategies such as polyandry, polygyny, and polygynandry. Monogamy was maintained across different habitats, rainfall, and a measure of insect abundance, territory distributions, and group sizes. Genetic mating strategy may be determined by other factors besides ecological conditions and group sizes, such as kinship structure (Nelson-Flower et al. 2011), genetic variability (Griffith et al. 2002), or lifespan (Arnold and Owens 2002). Perhaps, further work on apostlebirds to examine these alternate hypotheses may reveal the factors determining mating strategies in this species. To date, it remains unclear why such a small proportion of groups are employing polyandry, polygyny, and polygynandry and more interestingly, the consequence that these strategies may have on helping behaviors such as provisioning rates, as well as on long-term group stability, dyadic associations or breeding pair longevity.

## Data Accessibility

DNA sequences: GenBank accessions GU170386-GU170397, JQ838038 **-**JQ838044. Individual genotypes will be uploaded to DRYAD prior to publication. Individual group membership (including the nest where each offspring hatched): DRYAD. Readme.txt file (information on above two DRYAD files): DRYAD.
